# Good Vibrations: Calculating Excited-State Frequencies Using Ground-State Self-Consistent Field Models

**DOI:** 10.1021/acs.jctc.2c00672

**Published:** 2022-11-29

**Authors:** Ali Abou Taka, Hector H. Corzo, Aurora Pribram−Jones, Hrant P. Hratchian

**Affiliations:** † Department of Chemistry and Biochemistry and Center for Chemical Computation and Theory, University of California, Merced, California95343, United States; ‡ Combustion Research Facility, Sandia National Laboratories, Livermore, California94550, United States; § National Center for Computational Sciences, Oak Ridge Leadership Computing Facility, Oak Ridge National laboratory, Oak Ridge, Tennessee37831-6012, United States

## Abstract

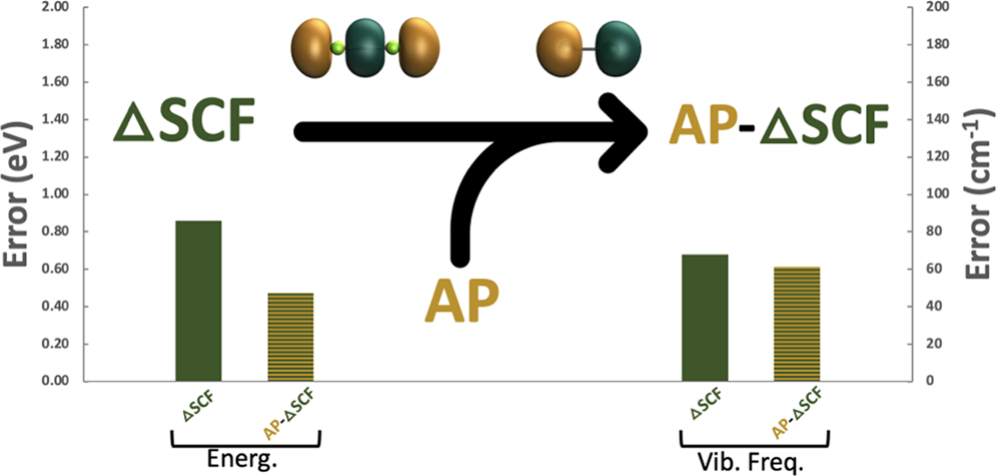

The use of Δ-self-consistent field (SCF) approaches for studying excited electronic states has received a renewed interest in recent years. In this work, the use of this scheme for calculating excited-state vibrational frequencies is examined. Results from Δ-SCF calculations for a set of representative molecules are compared with those obtained using configuration interaction with single substitutions (CIS) and time-dependent density functional theory (TD-DFT) methods. The use of an approximate spin purification model is also considered for cases where the excited-state SCF solution is spin-contaminated. The results of this work demonstrate that an SCF-based description of an excited-state potential energy surface can be an accurate and cost-effective alternative to CIS and TD-DFT methods.

## 1 Introduction

Electronic excited states play a critical role across science, including photochemistry, analytical chemistry, materials science, and biology.^1−11^ Computational chemistry serves a vital role in advancing such science by providing corroborating experimental interpretations through spectral simulations, predicting and interpreting excited-state properties, and providing insight into excited-state structure and dynamics through theoretical descriptions of excited-state potential energy surfaces (PESs).^12−20^ Despite numerous successful computational studies exploring photophysics and photochemistry in the literature, the development of new excited-state models and methods remains an active area of research in theoretical chemistry.^21−29^

Perhaps, the most conceptually straightforward and accurate quantum chemistry approaches for calculating excited electronic state wave functions are configuration interaction (CI)-based methods.^30−35^ More sophisticated multiconfigurational-SCF and multireference CI theories are also well-recognized models for calculating excited states.^36−38^ Unfortunately, in many cases, the computational expense of such models for moderate to large systems is prohibitively expensive.

Time-dependent density functional theory (TD-DFT) is an alternative single-reference excited-state model based on Kohn–Sham DFT.^39−42^ In TD-DFT, the many-body time-dependent Schrödinger equation is reformulated by a set of time-dependent single-particle equations with orbitals yielding the same time-dependent density. Due to its efficiency, black box nature, and inclusion of dynamic electron correlation, TD-DFT is the method of choice for most excited-state studies in molecular quantum chemistry.^43−58^

Among the methods based on Slater determinants, configuration interaction singles (CIS) and TD-DFT are the most popular candidates for investigations of excited states of large systems. In many cases, these models provide enough information for the characterization of excited-state systems, yet both models have notable limitations. CIS neglects important contributions to electron correlation.^59^ Since only single replacements are included in the determinantal basis, neither CIS nor TD-DFT can properly describe the electronic configuration of excited states at conical intersection processes.^60,61^ Most TD-DFT approximations give substantial errors for molecular excited states with extended π-systems.^62,63^

In recent years, the idea of self-consistent field (SCF) calculations for approximating excited electronic states using the maximum overlap method (MOM) has been reintroduced, including the initial maximum overlap method (IMOM).^22,23^ This approach has been used to explore various electronic and structural properties in molecules.^21−23,27,64−70^ In such methodologies, standard ground-state SCF algorithms are used to find a stationary point in the SCF space that maximizes the overlap with an initial guess set of occupied molecular orbitals (MOs). However, these low-cost approaches often suffer from a number of challenges, including convergence difficulties and variational collapse. Recently, the projected initial maximum overlap method (PIMOM), which uses a projection operator framework to determine a non-Aufbau metric for determining which molecular orbitals to occupy at each SCF cycle, was shown to overcome some of the challenges observed with alternative maximum overlap metrics.^26^ In fact, the projection-based framework provides a convenient connection to population analysis.^26^

In this work, we benchmark excited electronic state vibrational frequencies evaluated using SCF solutions that model excited-state electronic structures. To reliably locate excited-state wave functions as SCF solutions, we use the PIMOM.^26^ Although such Δ-SCF calculations are computationally feasible, the excited-state solution often exhibits (spin) symmetry breaking. Specifically, most singly excited states will result in open-shell results that exhibit spin contamination. While single-reference excited-state models can employ spin adaptation to overcome this challenge, the issue remains for the Δ-SCF approach. As we have shown in related contexts and as Herbert and co-workers recently reported for Δ-SCF treatments of excited-state energies, the broken-symmetry results can often be improved using an approximate projection (AP) measure.^27,71−73^ Some time ago, Hratchian and co-workers’ group derived and implemented analytical first and second energy derivatives of approximate projection (AP)-corrected energies.^74,75^ Using that theory, we further assess the impact of AP on excited-state geometries and vibrational frequencies.

## 2 Methods

In this section, the PIMOM and AP methods are briefly described. PIMOM is used to guide the SCF optimizer toward the desired solutions. The AP method is used to correct effects due to spin contamination in broken-symmetry SCF solutions.

### 2.1 Projected Initial Maximum Overlap Method

Using the Δ-SCF approach for studying electronic excitations requires the optimization of single-determinant SCF solutions that often are *not* minima in SCF space.^23^ For such calculations, unaltered SCF optimizers often experience variational collapse, thus failing to locate the desired state.^23^ PIMOM has recently been shown to be a reliable and robust scheme for optimizing SCF solutions resembling user-defined target single determinants.^26^

In the atomic orbital basis, the SCF equations are given by
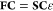
1where **F** is the Fock matrix, **C** is the matrix of MO coefficients, **S** is the atomic orbital overlap matrix, and ε is the orbital energy vector. Equation 1 is nonlinear and is solved iteratively. At each iteration, the Fock matrix is formed using the current density matrix, which is based on the choice of occupied MOs. Conventionally, this choice is made by the Aufbau principle.

Optimization to an SCF solution modeling an excited electronic state may be facilitated by imposing additional control over the spin–orbitals through symmetry restrictions, overlap matching, inclusion of additional constraints on the Lagrangian functions, or other means.^32,76−83^ Maximum overlap methods (MOMs) alter the standard SCF procedure by employing a modified-Aufbau rule whereby some metric of overlap, or agreement, between current-cycle Fock eigenstates and the occupied MOs of the user-defined target electronic structure is used to select occupied MOs. MOMs are particularly attractive members of this set of approaches as they often require only a minor modification of the existing SCF code infrastructure and can immediately be used with existing derivative and property theories in place for SCF wave functions/determinants. The choice for the non-Aufbau metric can result in very different performance by MOM calculations. Additionally, earlier MOMs tied the non-Aufbau metric evaluation at each SCF cycle to the previous iteration. More recently, Gill and co-workers have shown that a better approach is to tie the non-Aufbau metric to the *initial*, often user-defined, target electronic structure. Such an approach is termed the initial maximum overlap method (IMOM).

In an effort to understand and establish a physical interpretation for the performance of different non-Aufbau metrics, it was recently suggested that the non-Aufbau metric that arises naturally from a projection-based framework (*vide infra*) is both robust and effective at achieving SCF convergence and optimizing to the desired electronic structure. Using this particular non-Aufbau metric choice within an IMOM scheme gives rise to the specific model that we refer to as PIMOM. The PIMOM non-Aufbau metric is derived by beginning with the target system’s density projector
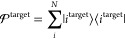
2In the MO basis of the current SCF cycle, eq 2 is given by
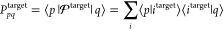
3which may be rewritten as
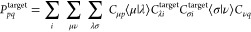
4where ⟨μ|ν⟩ = *S*_μν_ are the AO overlap matrix elements, and **C**^target^ is the target set of MO coefficients. In eq 4, the target density matrix in the AO basis may be expressed as *P*_μν_^target^ = ∑_*i*_*C*_μ*i*_^target^*C*_ν*i*_^target^. Thus, eq 4 may be written in the matrix form as
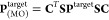
5where the subscript “MO” has been included to clearly indicate that the resulting density matrix is given in the current MO basis. The PIMOM scheme uses eq 5 to define the non-Aufbau metric as
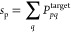
6*s*_p_ values will be used to dictate the order of the orbitals at each SCF cycle, where the orbitals with the largest *s*_p_ value will be occupied first. As with all MOMs, different SCF excited-state solutions are accessed by generating *s*_p_ values using a user-determined target solution.^23^

### 2.2 Approximate Projection Method

Spin contamination can affect the quality of excited-state energies and properties in Δ-SCF calculations due to the open-shell nature of most one-electron excitations.^27,77,84^ To address this potential impact, we have used the Yamaguchi approximate projection (AP) model with open-shell calculations described below.^85^ Our group has expanded the AP model to include analytical first and second derivatives.^74,75^ Several recent papers have demonstrated the effectiveness of this model and the conditions for which the AP model is suitable.^71,72,86,87^ In this subsection, we briefly outline the AP model.

To carry out AP calculations, two converged determinants are required: (1) an open-shell broken-symmetry state, i.e., the contaminated state, and (2) a spin-pure high-spin state that is taken to be degenerate with the state contaminating the broken-symmetry solution. Using the results of those two SCF solutions, the AP energy expression is given by
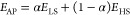
7where
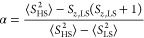
8In eqs 7 and 8, the “LS” subscript refers to the (broken-symmetry) low-spin solution and the “HS” subscript refers to the (spin-pure) high-spin state.

As discussed below, we have used a “contamination percentage” measure to identify excited states suffering from heavy spin contamination. The contamination percentage, α*, is given by
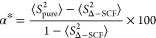
9The closer the value of α* to zero, the less impact spin contamination has on a given result. Thus, for systems with α* close to zero, AP will have mild or no effect on the calculated energies.

### 2.3 Computational Details

Δ-SCF results are reported below and compared with results obtained using CIS, TD-DFT, and experimental data. All Δ-SCF ground- and excited-state calculations were carried out using Becke’s three-parameter hybrid functional with Lee–Yang–Parr correlation (B3LYP)^88^ and the Hartree–Fock method.^89^ Four different basis sets have been used: 6-311G, 6-311++G(d,p),^90−94^ aug-cc-pVDZ, and aug-cc-pVTZ.^95−102^ Δ-SCF results were obtained using the PIMOM method. All electronic structure calculations were carried out using a local development version of the Gaussian suite of electronic structure programs.^103^

Molecular geometries for ground-state structures were optimized using standard methods,^104^ and the reported potential energy minima were verified using analytical second-derivative calculations. The ground-state minimum structures were used as a starting geometry for excited-state geometry optimizations using Δ-SCF, CIS, and TD-DFT methods. Excited-state optimized geometries were also verified using analytical second-derivative calculations. Initial electronic structure guesses for Δ-SCF calculations were generated by permuting MOs of the converged ground-state solution resembling the desired character of the target excited state. AP-Δ-SCF calculations and optimizations were carried out on the spin-contaminated systems and verified using analytical second-derivative calculations.^74^

## 3 Results and Discussion

Excited-state computation tools are expensive and somewhat limited compared to the ground-state toolbox, especially for polyatomic molecules.

The focus of the present study is the investigation of Δ-SCF for calculating excited-state vibrational frequencies. This work also demonstrates the use of PIMOM as an SCF driver. Additionally, we consider the AP model as a means for improving energies, geometries, and vibrational frequencies that may be affected by spin contamination. To assess the quality of the calculated results, we have compiled a data set chosen from available experimental gas-phase spectroscopy studies that can also be evaluated using the well-known CIS and TD-DFT methods for comparison.^105−114^ The data set contains various types of excited states and spin multiplicities.^115^

### 3.1 Adiabatic Excitation Energies

Δ-SCF methods^116,117^ have been successful in calculating vertical excitation energies, especially when the SCF optimizations have been guided by MOM algorithms.^22−25,84,118^Table 1 shows the adiabatic excitation energies (AEEs) calculated using TD-DFT and Δ-B3LYP with the two basis sets considered. TD-DFT performed better than Δ-B3LYP with mean absolute errors between 0.33 and 0.17 eV compared to 0.76 and 0.68 for Δ-B3LYP relative to experiment.

**Table 1:** Calculated Adiabatic Excitation Energies (eV) Using TD-DFT and Δ-DFT in Comparison with Experimental Values

		6-311G		6-311++G(d,p)	
sys	exp.	TD-DFT	Δ-DFT	TD-DFT	Δ-DFT
BH	2.87	2.75	1.67	2.74	1.69
BF	6.34	6.13	4.24	6.09	4.31
SiO	5.31	4.83	4.12	5.20	4.44
CO	8.07	7.51	6.21	7.95	6.60
N_2_	8.59	7.92	6.97	8.50	7.53
ScO	2.04	1.35	1.77	2.00	1.72
BeH	2.48	2.58	2.37	2.56	2.35
AsF	3.19	2.95	2.96	2.87	2.87
NH	3.70	3.98	3.64	3.90	3.61
CrF	1.01	1.47	1.44	1.25	1.23
CuH	2.91	3.35	2.46	2.98	2.70
Li_2_	1.74	1.93	1.09	1.93	1.07
Mg_2_	3.23	3.45	2.32	3.26	2.26
PH_2_	2.27	2.19	2.13	2.34	2.24
CH_2_S	2.03	2.04	1.64	2.06	1.67
C_2_H_2_	5.23	4.92	4.64	4.70	4.38
C_2_H_2_O_2_	2.72	2.21	1.93	2.42	2.12
HCP	4.31	3.91	3.74	3.86	3.60
HCN	6.48	6.02	5.70	5.95	5.59
C_3_H_4_O	3.21	2.98	2.64	3.15	2.78
CH_2_O	3.49	3.36	2.79	3.59	3.01
CCl_2_	2.14	-a	1.36	1.99	1.29
SiF_2_	5.34	4.85	3.79	5.31	3.96
MAE		0.33	0.76	0.17	0.68
RMSE		0.38	0.96	0.23	0.86

a TD-DFT failed to optimize the excited state.

**Figure 1:**
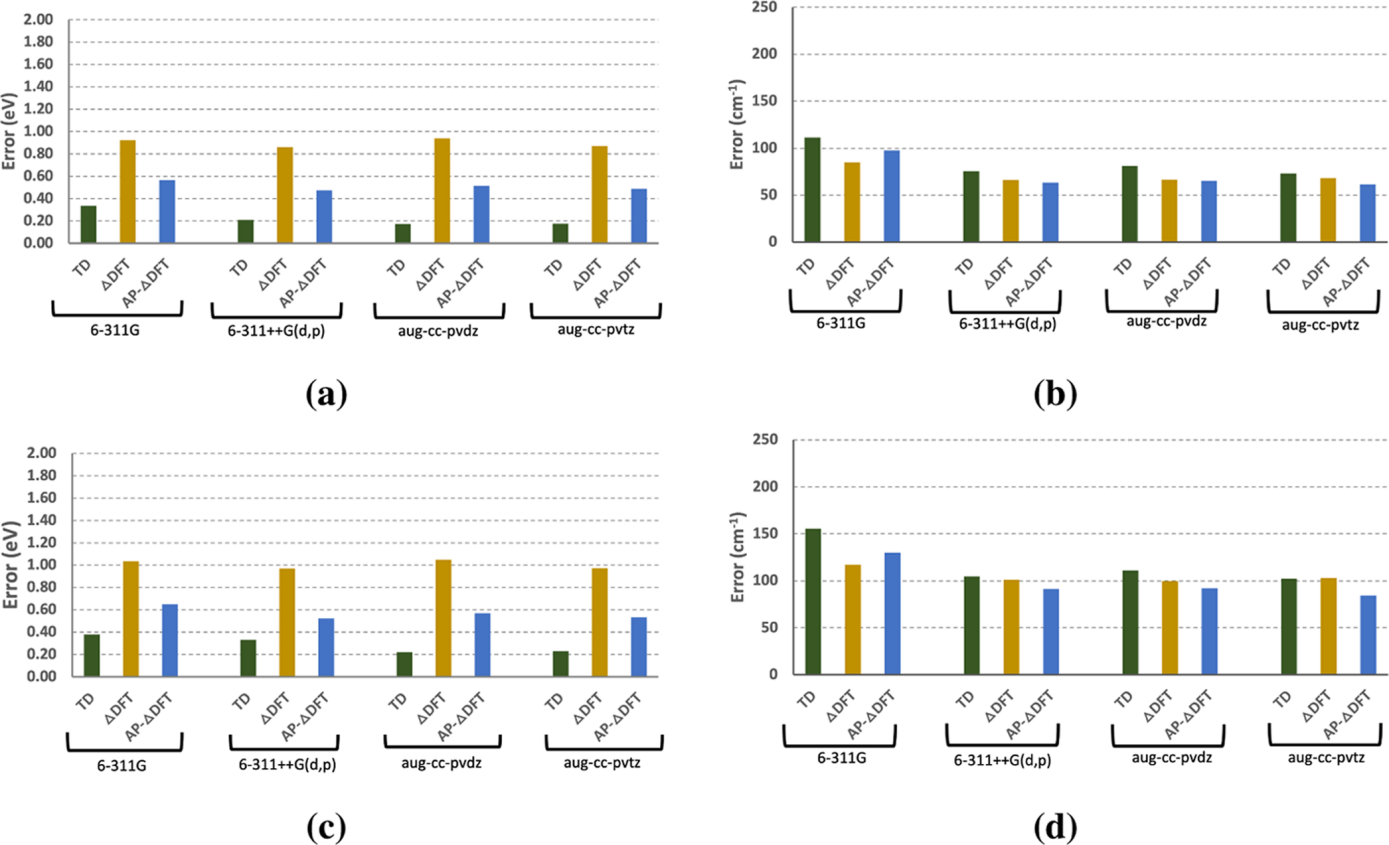
Mean absolute errors in (a) adiabatic excitation energies and (b) vibrational frequencies obtained using TD-DFT, Δ-DFT, and AP-Δ-DFT. root mean square errors (RMSE) in (c) adiabatic excitation energies and (d) vibrational frequencies obtained using TD-DFT, Δ-DFT, and AP-Δ-DFT are also shown.

Upon increasing the basis set from 6-311G to 6-311++G(d,p), the mean absolute error (MAE) of TD-DFT and Δ-B3LYP calculated AEEs decreased by 0.15 and 0.08 eV, respectively (Figure 1). This improvement can be explained by the addition of polarization and diffuse functions, which provides a better qualitative description of electronic excited states.^119^ A similar behavior is observed using correlation-consistent basis sets, for which the MAE for both TD- and Δ-B3LYP-based calculations decreased by 0.07 and 0.04 eV, respectively, upon increasing the basis set from aug-cc-pVDZ and aug-cc-pVTZ. This improvement shows milder basis set dependence than that observed with the Pople basis sets, which is not unexpected since both correlation-consistent basis sets use a larger number of polarization and diffuse functions.

**Figure 2:**
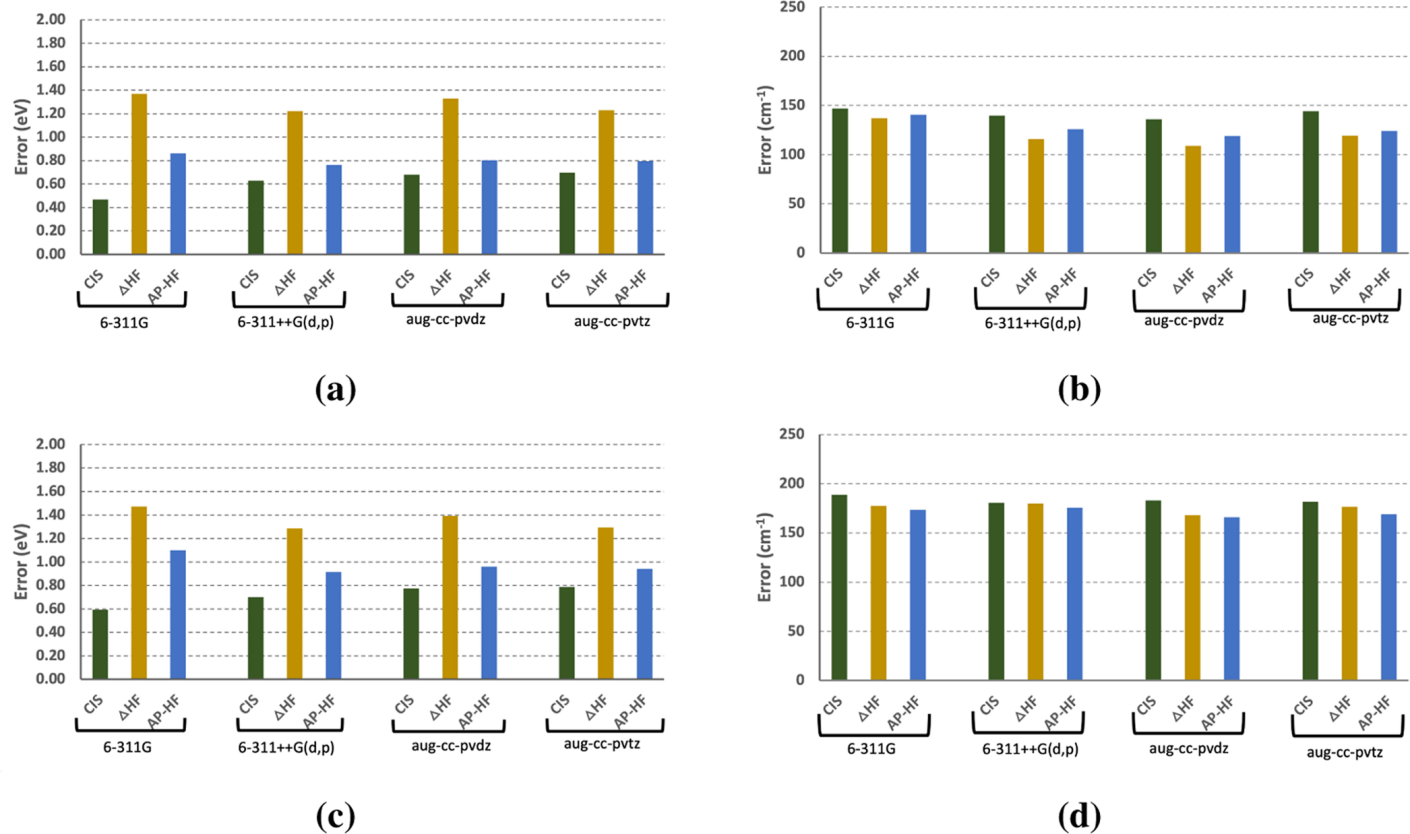
Mean absolute errors in (a) adiabatic excitation energies and (b) vibrational frequencies obtained using CIS, ΔHF, and AP-ΔHF. RMSE (a) adiabatic excitation energies and (b) vibrational frequencies are also reported for the same models.

In the case of CIS, the MAEs range between 0.41 and 0.55 eV, which is smaller than the MAE obtained using Δ-HF (1.00–1.14 eV), as reported in Table 2. Upon adding diffuse and polarization functions, unlike TD-DFT, the excitation energy accuracy decreased, where the MAE obtained using 6-311G is smaller than that of 6-311++G(d,p) by 0.14 eV (Figure 2). On the other hand, for correlation-consistent basis sets, a smaller difference is observed, 0.05 eV, favoring the larger basis set. Δ-HF showed an improved accuracy as the basis set size is increased, where MAE decreased by 0.14 eV from the smaller to the larger Pople basis set and decreased by 0.12 eV from the smaller to the larger correlation-consistent basis set.

**Table 2:** Calculated Adiabatic Excitation Energies (eV) Using CIS and Δ-HF in Comparison with Experimental Values

		6-311G		6-311++G(d,p)	
sys	exp.	CIS	Δ-HF	CIS	Δ-HF
BH	2.87	3.03	1.64	2.89	1.50
BF	6.34	6.49	4.39	6.54	4.51
SiO	5.31	5.23	2.90	6.09	3.74
CO	8.07	8.01	6.36	8.74	7.00
N_2_	8.59	8.65	7.25	9.45	8.06
ScO	2.04	2.30	1.60	2.07	2.05
BeH	2.48	2.78	2.64	2.76	2.64
AsF	3.19	3.83	3.57	3.76	3.44
NH	3.70	4.05	3.79	4.18	3.84
CrF	1.01	1.15	0.98	0.99	0.60
CuH	2.91	3.97	1.70	3.93	1.42
Li_2_	1.74	2.11	0.96	2.10	0.92
Mg_2_	3.23	3.59	2.69	3.34	2.46
PH_2_	2.27	2.33	2.20	2.68	2.38
CH_2_S	2.03	1.99	0.58	2.71	0.90
C_2_H_2_	5.23	4.68	4.07	4.49	3.71
C_2_H_2_O_2_	2.72	3.24	3.12	3.56	3.30
HCP	4.31	3.46	3.03	3.59	2.95
HCN	6.48	5.54	4.88	5.95	4.78
C_3_H_4_O	3.21	4.36	1.29	4.58	1.67
CH_2_O	3.49	3.99	1.51	4.10	1.66
CCl_2_	2.14	2.08	0.69	2.40	1.07
SiF_2_	5.34	5.69	3.97	5.96	4.09
MAE		0.41	1.07	0.55	0.97
RMSE		0.52	1.27	0.63	1.13

Absolute errors in TD-DFT and Δ-B3LYP AEEs are noticeably smaller than those found with CIS and Δ-HF, which is expected due to the correlation effects included in DFT. The AEEs obtained using Δ-SCF of the investigated systems here showed an underestimation, which can be attributed to several factors such as incomplete treatment of relaxation and correlation effects, as well as spin contamination. In general, the correct description of excited states requires a balanced treatment of orbital relaxation and correlation effects.

### 3.2 Excited-State Vibrational Analysis

Before exploring excited-state properties, it is important to evaluate the quality of the excited-state geometries and how well SCF-based geometries are compared to those determined using conventional excited-state models. First, in Table 3, we compare the bond lengths of the diatomic molecules. The bond lengths obtained using PIMOM-SCF are in very good agreement with those obtained with either TD-DFT and CIS. The absolute average difference of DFT methods is 0.057 Å without AP and 0.025 Å with AP. The average difference for Hartree–Fock (HF) and CIS is slightly higher than those with DFT but still very close with 0.083 Å without AP and 0.046 Å with AP. A similar behavior is observed for triatomic molecules (Table 4) where the differences in bond lengths and angles were small between all methods considered. For polyatomic molecules, the root-mean-square deviation (RMSD) is also computed to compare the geometries obtained with and without PIMOM-SCF (Table 5). The average deviations using both DFT and HF methods are low and range between 0.016 and 0.042 Å. Given the geometries obtained with PIMOM-SCF do not differ much from those with conventional excited-state methods, we then examined the quality of SCF-based excited-state vibrational frequencies.

**Table 3:** Absolute Difference in Bond Lengths (Å) for Diatomic Molecules Using (AP)Δ-B3LYP and TD-DFT and (AP)Δ-HF and CIS

sys.	Δ-B3LYP/TD-DFT	AP-Δ-B3LYP/TD-DFT	Δ-HF/CIS	AP-Δ-HF/CIS
BF	0.010	0.007	0.003	0.008
BH	0.023	0.014	0.011	0.004
CO	0.020	0.011	0.022	0.028
CuH	0.019	0.036	0.141	0.075
Li_2_	0.194	0.038	0.143	0.091
Mg_2_	0.041	0.075	0.225	0.086
N_2_	0.007	0.003	0.014	0.005
SiO	0.142	0.017	0.105	0.072
average difference	0.057	0.025	0.083	0.046

**Table 4:** Absolute Difference in Bond Lengths (Å) and Angles (°) for Triatomic Molecules Using (AP)Δ-B3LYP and TD-DFT and (AP)Δ-HF and CIS

sys.		Δ-B3LYP/TD-DFT	AP-Δ-B3LYP/TD-DFT	Δ-HF/CIS	AP-Δ-HF/CIS
CCl_2_	BL1	0.029	0.024	0.026	0.015
	BL2	0.029	0.024	0.026	0.015
	angle	8.603	7.601	3.611	0.879
HCN	BL1	0.007	0.003	0.004	0.014
	BL2	0.003	0.001	0.011	0.014
	angle	1.314	0.566	0.458	4.361
HCP	BL1	0.006	0.005	0.004	0.004
	BL2	0.002	0.002	0.003	0.002
	angle	2.021	2.141	3.611	0.981
SiF_2_	BL1	0.019	0.012	0.001	0.003
	BL2	0.019	0.012	0.001	0.003
	angle	1.140	0.904	1.466	2.260
average difference	BL1	0.015	0.011	0.009	0.009
	BL_2_	0.013	0.010	0.010	0.009
	angle	3.269	2.803	2.287	2.120

**Table 5:** Computed RMSD (Å) for Polyatomic Molecules Using (AP)Δ-B3LYP and TD-DFT and (AP)Δ-HF and CIS

sys.	Δ-B3LYP/TD-DFT	AP-Δ-B3LYP/TD-DFT	Δ-HF/CIS	AP-Δ-HF/CIS
C_2_H_2_O_2_	0.0048	0.0021	0.0056	0.006
C_3_H_4_O	0.0274	0.0246	0.0336	0.0348
CH_2_O	0.0238	0.0113	0.0671	0.0426
CH_2_S	0.0065	0.038	0.0618	0.0729
average deviation	0.016	0.019	0.042	0.039

The computed excited-state frequencies from all methods gave smaller relative percent errors than the relative percent errors of the adiabatic excitation energies (Tables 6 and 7). Unlike the computed excitation energies, the mean absolute errors for the excited-state frequencies obtained using Δ-SCF are less than those obtained using the TD-DFT or CIS methodologies by 11–28 cm^–1^, while the RMSEs are similar and range between 4 and 9 cm^–1^.

Δ-HF displayed an MAE that ranges between 112 and 139 cm^–1^, significantly higher than the MAE obtained using DFT, which attained an uppermost MAE of 79 cm^–1^. Nevertheless, Δ-HF performed similarly or better than CIS in all cases considered in this data set. For example, for the ν_3_(*a*′) mode of the CH_2_S, 1^1^A_2_ excited-state CIS resulted in a 30% error, much higher than the error resulting from Δ-HF (9%). Also, CIS gave large errors in describing the excited states of carbonyl compounds, such as C_3_H_8_O, CH_2_O, and (CHO)_2_. On the other hand, Δ-HF calculations gave much lower errors for most of the studied vibrational modes.

Unsurprisingly, TD-DFT and Δ-DFT performed better than CIS and Δ-HF for calculating excited-state vibrational frequencies. In general, the quality of calculated excited-state vibrational frequencies was better with Δ-B3LYP than with TD-B3LYP with all of the basis sets considered here. The lowest MAE was reported using 6-311++G(d,p). As such, we focus the remainder of our discussion on specific results using this basis set. In cases such as the 1 ^1^Σ_*u*_^+^ state of Mg_2_, the ν_2_(*a*_1_) mode of the 1^1^B_1_ of CCl_2_, and the ν_4_(*a*_1_) mode of the 1^1^A″ state of CH_2_O, Δ-B3LYP yielded remarkably more accurate vibrational frequencies than TD by 22, 36, and 14%, respectively. These results may be due to the incomplete TD-DFT treatment of the correlation effects in the excited states arising from nonvalence and degenerate orbitals.^120^ On the other hand, Δ-B3LYP errors for the 1^1^Σ_u_^+^ of Li_2_ and 1^1^Π of CO were greater than TD-B3LYP by 16 and 10%, respectively. These results may be connected to orbital relaxation in the Δ-B3LYP calculation.

The MAE of TD ranges between 73 and 107 cm^–1^ lower than that of CIS, which ranges between 139 and 142 cm^–1^. These results may be attributed to the exchange–correlation effects in DFT. This gives DFT a clear advantage over the CIS and HF methods.

Basis set effects are also less significant in the accuracy of the calculated vibrational frequencies than in adiabatic excitation energy calculations. In the case of Pople-style basis sets, the addition of diffuse and polarization functions lowered the excited-state fundamental vibrational frequency MAE by 34 and 17 cm^–1^ for TD- and Δ-B3LYP, respectively. Correlation-consistent basis sets showed similar behavior, with an improvement in accuracy of 8 cm^–1^ for both TD- and Δ-B3LYP, respectively. The lowest MAEs of 62 cm^–1^ with Δ-B3LYP and 73 cm^–1^ with TD were given by calculations employing the 6-311++G(d,p) basis set.

### 3.3 Spin Contamination

In many cases, excited states obtained using the Δ-SCF approach are spin-contaminated, motivating the examination of spin purification methods.^27,85,121−123^ As mentioned above, we have used the AP model of Yamaguchi and co-workers,^85^ for which analytic first and second derivatives have been reported.^71,72,74,75,86,87^ Using eq 9 and a threshold of 5% spin contamination, 17 cases out of 25 were identified as being spin-contaminated with Δ-DFT and explored further using the AP model (Table 6). It is important to note that the AP model is expected to behave well only for situations where the spin-contaminated state has only one higher spin contaminant to be projected out. With this in mind, we identified C_2_H_2_ and CO as systems inappropriate for this AP approach. Using HF with all of the basis sets considered, the triplet state of C_2_H_2_ exhibited geometric symmetry breaking, *C*_2*h*_ to *C*_*s*_, and the different symmetries of the low- and high-spin states caused difficulties in AP convergence. Using HF/6-311G, CO was also excluded since the triplet solution showed significant spin contamination. For all other cases, AP showed a similar performance with all basis sets considered. Thus, we limit our discussion of spin purification results to the 6-311++G(d,p) basis set. Full details obtained using all model chemistries considered in this work are provided in the Supporting Information (Tables S1–S16).

**Table 6:** Adiabatic Excitation Energies before and after Approximate Projection on Systems with Spin Contamination above 5%^a^

sys.	exp.	TD	Δ-B3LYP	AP−Δ-B3LYP	CIS	Δ-HF	AP−Δ-HF
BH	2.87	2.74	1.69	2.30	2.89	1.50	2.68
BF	6.34	6.09	4.31	5.26	6.54	4.51	6.54
SiO	5.31	5.20	4.44	4.83	6.09	3.74	3.97
CO	8.07	7.95	6.60	7.37	8.74	7.00	8.63
N_2_	8.59	8.50	7.53	8.03	9.45	8.06	8.83
CuH	2.91	2.98	2.70	3.00	3.93	1.42	1.93
Li_2_	1.74	1.93	1.07	1.21	2.10	0.92	1.47
CCl_2_	2.14	1.99	1.29	1.81	2.40	1.07	2.18
CH_2_S	2.03	2.06	1.67	1.75	2.71	0.90	0.92
Mg_2_	3.23	3.26	2.26	2.70	3.34	2.46	3.79
C_2_H_2_O	2.72	2.42	2.12	2.31	3.56	3.30	3.31
HCP	4.31	3.86	3.65	3.83	3.59	2.95	3.26
CH_2_O	3.49	3.59	3.01	3.17	4.10	1.66	1.76
C_3_H_4_O	3.21	3.15	2.78	2.87	4.58	1.67	1.73
SiF_2_	5.34	5.31	3.96	4.72	5.96	4.09	5.92
HCN	6.48	5.95	5.59	5.85	5.95	4.78	5.23
C_2_H_2_	5.23	4.70	4.38	4.61	4.49	3.71	
MAE		0.17	0.86	0.47	0.63	1.22	0.76
RMSE		0.22	0.97	0.52	0.70	1.29	0.91

a 6-311++G(d,p) basis set was used.

In agreement with recent work from Herbert and co-workers,^27^ our results show that the AP model yields significant corrections to energies for all model chemistries considered. As shown in Table 6 and Figures 1 and 2, the MAE for Δ-SCF methods incorporating AP corrections decreased by ∼0.4 eV for Δ-DFT and ∼0.5 eV for Δ-HF model chemistries. For the specific cases of BF and SiF_2_, AP-Δ-DFT reduced the error by 0.95 and 0.76 eV, respectively (using the 6-311++G(d,p) basis set). Similar behavior has been observed for the AP-Δ-HF method, where the error of BF and SiF_2_ dropped by 1.63 and 0.67 eV, respectively. On the other hand, it is well known that Δ-SCF excitation energies for open-shell singlets, despite the spin contamination, are often unexpectedly accurate.^22−24^ This was observed in the cases of CuH where the error with AP dropped from 0.21 to 0.09 eV, and for CH_2_S where the error decreased from 0.36 to 0.28 eV. We note that while these last examples demonstrate smaller energy corrections with AP than the more significant cases listed earlier, they are nevertheless meaningful energy corrections (Table 7).

**Table 7:** Vibrational Frequencies Obtained Using the 6-311++G(d,p) Basis Set before and after Approximate Projection^a^

sys.	state	exp.	TD	Δ-B3LYP	AP-Δ-B3LYP
BH	1^1^Π	2251	2363	2510	2421
BF	1^1^Π	1265	1224	1262	1256
SiO	1^1^Π	853	884	881	809
CO	1^1^Π	1518	1539	1693	1596
N_2_	1^1^Πg	1694	1737	1791	1765
CuH	2^1^Σ^+^	1698	1650	1637	1623
Li_2_	1^1^Σ_*u*_^+^	255	261	208	267
Mg_2_	1^1^Σ_*u*_^+^	191	156	191	162
CH_2_S^b^	1^1^*A*_2_	799	801	782	795
		820	896	836	822
		1316	1372	1351	1355
		3034	3127	3112	3101
		3081	3240	3228	3217
C_2_H_2_	1^1^*A*_*u*_	1048	1092	1103	1100
		1385	1433	1420	1419
C_2_H_2_O_2_^c^	1^1^*A*_*u*_	233	251	243	241
		379	386	400	392
		509	519	516	517
		720	779	758	762
		735	780	772	767
		952	971	974	965
		1172	1197	1224	1211
		1196	1239	1242	1238
		1281	1528	1426	1404
		1391	1572	1556	1564
		2809	2966	3003	2979
HCP	1^1^*A*″	567	694	716	712
		951	957	947	947
HCN	1^1^*A*″	941	983	985	991
		1496	1531	1509	1528
C_3_H_4_O^d^	1^1^*A*″	250	261	240	258
		333	295	292	292
		488	504	498	501
		582	508	514	502
		644	709	625	679
		909	934	941	950
		1266	1094	1087	1080
		1133	1376	1313	1307
CH_2_O^e^	1^1^*A*″	683	575	698	634
		899	891	894	914
		1177	1300	1247	1215
		1321	1358	1301	1314
		2851	2987	2954	2973
		2968	3085	3048	3070
CCl_2_^f^	1^1^*B*_1_	303	192	300	301
		634	590	638	620
SiF_2_^g^	1^1^*B*_1_	252	233	242	240
		860	672	748	723
		984	768	861	835
MEA			77	66	63
RMSE			105	101	92

a Experimental results are taken from ref (105) for diatomic and from ref (106) for polyatomic molecules unless otherwise stated.

b Experimental data from ref (108).

c Experimental data from ref (109).

d Experimental data from refs (110) and (111).

e Experimental data from ref (112).

f Experimental data from ref (113).

g Experimental data from ref (114)

The noted improvement in Δ-SCF excited-state energies with AP spin purification was not as apparent with excited-state fundamental frequencies (Figures 1 and 2). AP provided modest improvements for calculated vibrational frequencies when diffuse and/or polarization functions are included in the basis sets. In such cases, the MAE decreased by ∼4 cm^–1^ and the RMSE decreased by ∼11 cm^–1^. However, AP calculations using the 6-311G basis set resulted in MAE and RMSE *increases* by ∼12 cm^–1^ relative to the corresponding spin-contaminated results. Interestingly, the MAE of excited-state frequencies obtained by AP-Δ-HF increased relative to Δ-HF by ∼4 cm^–1^, while the RMSE decreased by ∼10 cm^–1^ when using AP. This suggests that, while the mean error slightly increased with spin purification, the spread of errors noticeably decreased using AP.

Overall, it appears that spin purification is a useful tool for improving the general performance of Δ-SCF calculations when studying excited states. Importantly, we note that both Δ-DFT and AP-Δ-DFT perform better than TD-DFT in predicting vibrational frequencies relative to the experiment (see Figure 1). AP-Δ-SCF is expected to perform comparably to Δ-SCF methods, but some caution is warranted based on the system under investigation. The spin correction results shown here are expected and agree with a previous study suggesting that spin projection often does not result in large structural changes but can give meaningful changes in energy.^87^

### 3.4 Initial Guess Generation

The choice of the SCF initial guess determinant is crucial to the success of MOM calculations. An orbital permutation from the reference ground state to match the excited state in nature and symmetry often suffices as an initial guess for the PIMOM framework. Indeed, that approach led to successful outcomes for nearly all of the calculations included in this work.

However, that straightforward and intuitive approach is not always successful. One such case was the first ^1^Π excited state of SiO. TD-DFT shows three configurations involved in representing this excitation, with an amplitude of 0.17162 for the HOMO → LUMO+1 determinant, 0.67339 for the HOMO → LUMO determinant, and −0.12437 for the HOMO–3 → LUMO determinant. Generating a PIMOM initial guess by permuting the highest occupied molecular orbital (HOMO) with the lowest unoccupied molecular orbital (LUMO) or the HOMO with LUMO+1 led to an SCF excited-state representation giving an excitation energy of 4.44 eV. However, permuting the HOMO-3 with the LUMO led to an approximate excited state located 8.20 eV above the ground state. Clearly, the last altered determinant did not lead to the desired result. However, both of the first two permutations led to the correct state.

An alternative also explored for this work involved carrying out a single-point TD-DFT energy calculation followed by a natural transition orbital (NTO) transformation corresponding to the state of interest.^124^ Using the resulting NTOs to define the initial guess orbitals led us to the correct state in all cases, including the challenging case of SiO. We suggest the NTO model as an approach for generating initial states, particularly in instances where there is no clear one-electron transition in the canonical molecular orbital basis. Further examination of possible approaches for selecting initial target determinants remains an area of study for us (and others).

## 4 Conclusions

In this paper, we presented a Δ-SCF approach using the PIMOM framework to calculate AEEs and vibrational frequencies. Although TD-DFT and CIS provided slightly better energetics than PIMOM, the excited-state vibrational frequencies obtained with Δ-SCF were in better agreement with experimental results than either CIS or TD-DFT. The AP model improved the AEEs for both HF and DFT and it did not have a significant effect on the vibrational frequencies.

Since SCF calculations are more affordable than available excited-state methods, especially for large systems, PIMOM presents a viable computational approach for modeling excited-state molecular properties with ground-state computational cost. While AP-corrected second derivatives have a minimal effect on calculated frequencies, this work demonstrates the significance of using the AP model to correct AEEs. Given the results shown in this work, the AP-Δ-SCF approach offers comparable performance to single-reference excited-state models such as CIS and TD-DFT with a lower computational cost.

We note that most of the test cases included in this work are relatively small. However, we believe that this initial benchmark set provides for a reasonable examination of using Δ-SCF methods for evaluating excited-state energies and vibrational frequencies. Furthermore, this work demonstrates the usefulness of SCF driver methods such as PIMOM for facilitating such calculations. Future work will further explore the use of PIMOM-based Δ-SCF calculations for studying electronic excited-state chemistry.

## Supporting Information Available

Excitation energies and vibrational frequencies (Tables S1–S16) and all optimized structures (PDF)

## Notes

This manuscript has been authored by UT-Battelle, LLC, under contract DE-AC05-00OR22725 with the U.S. Department of Energy (DOE). The U.S. government retains and the publisher, by accepting the article for publication, acknowledges that the U.S. government retains a nonexclusive, paid-up, irrevocable, worldwide license to publish or reproduce the published form of this manuscript, or allow others to do so, for U.S. government purposes. DOE will provide public access to these results of federally sponsored research in accordance with the DOE Public Access Plan (http://energy.gov/downloads/doe-public-access-plan).
