# Supplementary material for: Good Vibrations: Calculating Excited-State Frequencies Using Ground-State Self-Consistent Field Models
Source: J Chem Theory Comput. 2022 Nov 29;18(12):7286–97. doi: 10.1021/acs.jctc.2c00672 (PMC9753584; doi:10.1021/acs.jctc.2c00672)
Supplement: Supplementary file 1 — ct2c00672_si_001.pdf [file ct2c00672_si_001.pdf]

Supporting Information: Good Vibrations: Calculating Excited State Frequencies Using Ground State Self-Consistent Field Models

Ali Abou Taka,[†,‡] Hector H. Corzo,[†,‡] Aurora Pribram–Jones,[*,†] and Hrant P. Hratchian[*,†]

†*Department of Chemistry and Biochemistry and Center for Chemical Computation and Theory, University of California, Merced, California 95343, USA*

‡*(A.A.T. and H.H.C.) These authors contributed equally to this work.*

E-mail: apj@ucmerced.edu; hhratchian@ucmerced.edu

Contents

Supporting Information Available **S-2**

1 Tables **S-2**

2 Geometries **S-18**

Supporting Information Available

1 Tables

Table S1: Excitation energies obtained using Δ-B3LYP.

System	6-311G	6-311++G(d,p)	aug-cc-PVDZ	aug-cc-PVTZ	Exp.
**BH**	1.67	1.69	1.69	1.68	**2.87**
**BF**	4.24	4.31	4.29	4.34	**6.34**
**SiO**	4.12	4.44	3.78	4.39	**5.31**
**CO**	6.21	6.60	6.56	6.6	**8.07**
**N_2**	6.97	7.53	7.44	7.57	**8.59**
**ScO**	1.77	1.72	1.71	1.38	**2.04**
**BeH**	2.37	2.35	2.36	2.33	**2.48**
**AsF**	2.96	2.87	2.96	3.37	**3.19**
**NH**	3.64	3.61	3.61	3.59	**3.70**
**CrF**	1.44	1.23	1.22	1.23	**1.01**
**CuH**	2.46	2.70	2.61	2.65	**2.91**
**Li_2**	1.09	1.07	1.07	1.06	**1.74**
**CCl_2**	1.36	1.29	1.35	1.27	**2.14**
**CH_2S**	1.64	1.67	1.64	1.64	**2.03**
**Mg_2**	2.32	2.26	2.27	2.27	**3.23**
**PH_2**	2.13	2.24	2.24	2.22	**2.27**
**$C_2H_2O_2$**	1.93	2.12	2.09	2.11	**2.72**
**HCP**	3.74	3.60	3.50	3.56	**4.31**
**CH_2O**	2.79	3.01	2.96	3.00	**3.49**
**C_3H_4O**	2.64	2.78	2.74	2.77	**3.21**
**SiF_2**	3.79	3.96	3.94	3.95	**5.34**
**HCN**	5.7	5.59	5.45	5.57	**6.48**
**C_2H_2**	4.64	4.38	4.22	4.38	**5.23**
**MAE**	**0.78**	**0.68**	**0.74**	**0.70**	
**RMSE**	**0.96**	**0.84**	**0.90**	**0.85**	

Table S2: Excitation energies obtained using TDDFT.

System	6-311G	6-311++G(d,p)	aug-cc-PVDZ	aug-cc-PVTZ	Exp.
**BH**	2.75	2.74	2.67	2.69	**2.87**
**BF**	6.13	6.09	6.08	6.09	**6.34**
**SiO**	4.83	5.20	4.54	5.16	**5.31**
**CO**	7.51	7.95	7.90	7.96	**8.07**
**N_2**	7.92	8.50	8.41	8.56	**8.59**
**ScO**	1.35	2.00	1.97	1.98	**2.04**
**BeH**	2.58	2.56	2.58	2.57	**2.48**
**AsF**	2.95	2.87	2.96	3.03	**3.19**
**NH**	3.98	3.90	3.87	3.86	**3.70**
**CrF**	1.47	1.25	1.22	1.25	**1.01**
**CuH**	3.35	2.98	2.92	2.96	**2.91**
**Li_2**	1.93	1.93	1.93	1.93	**1.74**
**CCl_2**	X	1.99	1.71	1.96	**2.14**
**CH_2S**	2.04	2.06	2.04	2.07	**2.03**
**Mg_2**	3.45	3.26	3.32	3.26	**3.23**
**PH_2**	2.19	2.34	2.33	2.34	**2.27**
**$C_2H_2O_2$**	2.21	2.42	2.39	2.42	**2.72**
**HCP**	3.91	3.86	3.74	3.82	**4.31**
**CH_2O**	3.36	3.59	3.54	3.60	**3.49**
**C_3H_4O**	2.98	3.15	3.11	3.16	**3.21**
**SiF_2**	4.85	5.31	5.25	5.31	**5.34**
**HCN**	6.02	5.95	5.82	5.96	**6.48**
**C_2H_2**	4.92	4.70	4.55	4.73	**5.23**
**MAE**	**0.34**	**0.17**	**0.24**	**0.17**	
**RMSE**	**0.38**	**0.22**	**0.33**	**0.23**	

Table S3: Excitation energies obtained using Δ-HF.

System	6-311G	6-311++G(d,p)	aug-cc-PVDZ	aug-cc-PVTZ	Exp.
**BH**	1.64	1.5	1.68	1.47	**2.87**
**BF**	4.39	4.51	4.47	4.55	**6.34**
**SiO**	2.90	3.74	3.52	3.68	**5.31**
**CO**	6.36	7.00	6.95	6.99	**8.07**
**N_2**	7.25	8.06	6.77	8.08	**8.59**
**ScO**	1.60	2.05	2.02	2.04	**2.04**
**BeH**	2.64	2.64	2.62	2.61	**2.48**
**AsF**	3.57	3.44	3.54	3.66	**3.19**
**NH**	3.79	3.84	3.83	3.80	**3.70**
**CrF**	0.98	0.60	0.59	0.60	**1.01**
**CuH**	1.7	1.42	1.38	1.37	**2.91**
**Li_2**	0.96	0.92	0.92	0.94	**1.74**
**CCl_2**	0.69	1.07	0.93	1.05	**2.14**
**CH_2S**	0.58	0.90	0.88	0.77	**2.03**
**Mg_2**	2.69	2.46	2.47	2.45	**3.23**
**PH_2**	2.20	2.38	2.35	2.34	**2.27**
**$C_2H_2O_2$**	3.12	3.30	3.26	3.27	**2.72**
**HCP**	3.03	2.95	2.72	2.76	**4.31**
**CH_2O**	1.51	1.66	1.98	2.00	**3.49**
**C_3H_4O**	1.29	1.67	1.61	1.64	**3.21**
**SiF_2**	3.97	4.09	4.08	4.07	**5.34**
**HCN**	4.88	4.78	4.62	4.73	**6.48**
**C_2H_2**	4.07	3.71	3.53	3.68	**5.23**
**MAE**	**1.07**	**1.00**	**1.09**	**0.97**	
**RMSE**	**1.27**	**1.19**	**1.29**	**1.13**	

Table S4: Excitation energies obtained using CIS.

System	6-311G	6-311++G(d,p)	aug-cc-PVDZ	aug-cc-PVTZ	Exp.
**BH**	3.03	2.89	2.85	2.86	**2.87**
**BF**	6.49	6.54	6.51	6.57	**6.34**
**SiO**	5.23	6.09	4.17	6.09	**5.31**
**CO**	8.01	8.74	8.69	8.76	**8.07**
**N_2**	8.56	9.45	9.38	9.53	**8.59**
**ScO**	2.30	2.07	2.10	2.05	**2.04**
**BeH**	2.78	2.76	2.74	2.73	**2.48**
**AsF**	3.83	3.76	3.84	3.95	**3.19**
**NH**	4.05	4.18	4.18	4.19	**3.70**
**CrF**	1.15	0.99	0.93	0.99	**1.01**
**CuH**	3.97	3.93	3.92	3.93	**2.91**
**Li_2**	2.11	2.10	2.10	2.13	**1.74**
**CCl_2**	2.08	2.40	2.15	2.39	**2.14**
**CH_2S**	1.99	2.71	2.70	2.61	**2.03**
**Mg_2**	3.59	3.34	3.35	3.34	**3.23**
**PH_2**	2.33	2.68	2.72	2.78	**2.27**
**$C_2H_2O_2$**	3.24	3.56	3.55	3.59	**2.72**
**HCP**	3.46	3.59	3.35	4.24	**4.31**
**CH_2O**	3.99	4.10	4.39	4.44	**3.49**
**C_3H_4O**	4.36	4.58	4.54	4.58	**3.21**
**SiF_2**	5.69	5.96	5.93	5.94	**5.34**
**HCN**	5.54	5.95	5.50	5.65	**6.48**
**C_2H_2**	4.68	4.49	4.34	4.68	**5.23**
**MAE**	**0.41**	**0.55**	**0.60**	**0.55**	
**RMSE**	**0.52**	**0.63**	**0.70**	**0.65**	

Table S5: Excitation energies obtained using the 6-311G basis set before and after approximate projection.

System	Exp.	TDDFT	Δ-B3LYP	AP-Δ-B3LYP	CIS	Δ-HF	AP-Δ-HF
**BH**	**2.48**	2.75	1.67	2.26	3.03	1.64	2.89
**BF**	**6.34**	6.13	4.24	5.17	6.49	4.39	6.37
**SiO**	**5.31**	4.83	4.12	4.40	5.23	2.90	3.05
**CO**	**8.07**	7.51	6.21	6.92	8.01	6.36	-
**N_2**	**8.59**	7.92	6.97	7.45	8.56	7.25	7.99
**CuH**	**2.91**	3.35	2.46	2.80	3.97	1.70	2.61
**Li_2**	**1.74**	1.93	1.09	1.25	2.11	0.96	1.55
**CCl_2**	**2.14**		1.36	1.92	2.08	0.69	1.89
**CH_2S**	**2.03**	2.04	1.64	1.71	1.99	0.58	0.59
**Mg_2**	**3.23**	3.45	2.32	2.79	3.59	2.69	4.07
**C2H2O**	**2.72**	2.21	1.93	2.14	3.24	3.12	3.56
**HCP**	**4.31**	3.91	3.74	3.97	3.46	3.03	3.52
**CH_2O**	**3.49**	3.36	2.89	2.94	3.99	1.51	1.59
**C_3H_4O**	**3.21**	2.98	2.54	2.64	4.36	1.29	1.33
**SiF_2**	**5.34**	4.85	3.79	4.38	5.69	3.97	5.52
**HCN**	**6.48**	6.02	5.70	6.01	5.54	4.88	5.47
**C_2H_2**	**5.23**	4.92	4.64	5.92	4.68	4.07	-
**MEA**		**0.34**	**0.92**	**0.57**	**0.47**	**1.37**	**0.86**
**RMSE**		**0.38**	**1.04**	**0.65**	**0.59**	**1.47**	**1.10**

Table S6: Excitation energies obtained using the 6-311++G(d,p) basis set before and after approximate projection.

System	Exp.	TDDFT	Δ-B3LYP	AP-Δ-B3LYP	CIS	Δ-HF	AP-Δ-HF
**BH**	**2.87**	2.74	1.69	2.30	2.89	1.50	2.68
**BF**	**6.34**	6.09	4.31	5.26	6.54	4.51	6.54
**SiO**	**5.31**	5.20	4.44	4.83	6.09	3.74	3.97
**CO**	**8.07**	7.95	6.60	7.37	8.74	7.00	8.63
**N_2**	**8.59**	8.50	7.53	8.03	9.45	8.06	8.83
**CuH**	**2.91**	2.98	2.70	3.00	3.93	1.42	1.93
**Li_2**	**1.74**	1.93	1.07	1.21	2.10	0.92	1.47
**CCl_2**	**2.14**	1.99	1.29	1.81	2.40	1.07	2.18
**CH_2S**	**2.03**	2.06	1.67	1.75	2.71	0.90	0.92
**Mg_2**	**3.23**	3.26	2.26	2.70	3.34	2.46	3.79
**C2H2O**	**2.72**	2.42	2.12	2.31	3.56	3.30	3.31
**HCP**	**4.31**	3.86	3.60	3.83	3.59	2.95	3.26
**CH_2O**	**3.49**	3.59	3.01	3.17	4.10	1.66	1.76
**C_3H_4O**	**3.21**	3.15	2.78	2.87	4.58	1.67	1.73
**SiF_2**	**5.34**	5.31	3.96	4.72	5.96	4.09	5.92
**HCN**	**6.48**	5.96	5.59	5.85	5.95	4.78	5.23
**C_2H_2**	**5.23**	4.70	4.38	4.61	4.49	3.71	-
**MEA**		**0.17**	**0.86**	**0.47**	**0.63**	**1.22**	**0.76**
**RMSE**		**0.22**	**0.97**	**0.52**	**0.70**	**1.29**	**0.91**

Table S7: Excitation energies obtained using the aug-cc-PVDZ basis set before and after approximate projection.

System	Exp.	TDDFT	Δ-B3LYP	AP-Δ-B3LYP	CIS	Δ-HF	AP-Δ-HF
**BH**	**2.48**	2.67	1.69	2.22	2.85	1.68	2.62
**BF**	**6.34**	6.08	4.29	5.25	6.51	4.47	6.51
**SiO**	**5.31**	4.54	3.78	4.71	4.17	3.52	3.78
**CO**	**8.07**	7.90	6.56	7.33	8.69	6.95	8.55
**N_2**	**8.59**	8.41	7.44	7.94	9.38	6.77	8.76
**CuH**	**2.91**	2.92	2.61	2.90	3.92	1.38	1.89
**Li_2**	**1.74**	1.93	1.07	1.20	2.10	0.92	1.46
**CCl_2**	**2.14**	1.71	1.35	1.86	2.15	0.93	2.01
**CH_2S**	**2.03**	2.04	1.64	1.72	2.70	0.88	0.89
**Mg_2**	**3.23**	3.32	2.27	2.71	3.35	2.47	3.80
**C2H2O**	**2.72**	2.39	2.09	2.28	3.55	3.26	3.65
**HCP**	**4.31**	3.74	3.50	3.67	3.35	2.72	2.99
**CH_2O**	**3.49**	3.54	2.96	3.12	4.39	1.98	2.08
**C_3H_4O**	**3.21**	3.11	2.74	2.83	4.54	1.61	1.67
**SiF_2**	**5.34**	5.25	3.94	4.7	5.93	4.08	5.94
**HCN**	**6.48**	6.44	5.45	5.71	5.50	4.62	5.07
**C_2H_2**	**5.23**	4.55	4.22	4.44	4.34	3.53	-
**MEA**		**0.22**	**0.94**	**0.52**	**0.68**	**1.33**	**0.80**
RMSE		**0.30**	**1.05**	**0.57**	**0.77**	**1.39**	**0.96**

Table S8: Excitation energies obtained using the aug-cc-PVTZ basis set before and after approximate projection.

System	Exp.	TDDFT	Δ-B3LYP	AP-Δ-B3LYP	CIS	Δ-HF	AP-Δ-HF
**BH**	**2.48**	2.69	1.68	2.21	2.86	1.47	2.62
**BF**	**6.34**	6.09	4.34	5.28	6.57	4.55	6.58
**SiO**	**5.31**	5.16	4.39	4.78	6.09	3.68	3.94
**CO**	**8.07**	7.96	6.60	7.37	8.76	6.99	8.62
**N_2**	**8.59**	8.56	7.57	8.05	9.53	8.08	8.84
**CuH**	**2.91**	2.96	2.65	2.95	3.93	1.37	1.90
**Li_2**	**1.74**	1.93	1.06	1.21	2.13	0.94	1.48
**CCl_2**	**2.14**	1.96	1.27	1.75	2.39	1.05	2.10
**CH_2S**	**2.03**	2.07	1.64	1.71	3.34	0.77	0.77
**Mg_2**	**3.23**	2.34	2.27	2.79	2.78	2.45	3.70
**C2H2O**	**2.72**	2.42	2.11	2.3	3.59	3.27	3.65
**HCP**	**4.31**	3.82	3.56	3.72	4.24	2.76	2.99
**CH_2O**	**3.49**	3.60	3.00	3.15	4.44	2.00	2.10
**C_3H_4O**	**3.21**	3.16	2.77	2.86	4.58	1.64	1.70
**SiF_2**	**5.34**	5.31	3.95	4.73	5.94	4.07	5.97
**HCN**	**6.48**	5.95	5.57	5.82	5.65	4.73	5.14
**C_2H_2**	**5.23**	5.95	4.38	4.59	4.68	3.68	-
**MEA**		**0.23**	**0.87**	**0.49**	**0.70**	**1.23**	**0.79**
**RMSE**		**0.32**	**0.97**	**0.53**	**0.79**	**1.29**	**0.94**

Table S9: Vibrational frequencies obtained using the 6-311G basis set before and after approximate projection.

System	State	Exp.	CIS	Δ-HF	Δ-HF-AP-PIMOM
**BH**	$1^1\Pi$	2251	2442	2441	2191
**BF**	$1^1\Pi$	1265	1169	1171	1162
**SiO**	$1^1\Pi$	853	738	722	710
**N_2**	$1^1\Pi g$	1694	1774	1770	1736
**CuH**	$2^1\Sigma^+$	1698	1734	1589	1588
**Li_2**	$1^1\Sigma_u^+ +$	255	273	235	322
**Mg_2**	$1^1\Sigma_u^+$	191	254	184	120
**CH_2S**	1^1A_2	799	811	725	672
		820	845	901	897
		1316	1393	1473	1461
		3034	3140	3267	3241
		3081	3275	3418	3389
**$C_2H_2O_2$**	1^1A_u	233	263	256	240
		379	440	434	429
		509	572	557	556
		720	871	893	885
		735	910	904	936
		952	1086	1067	1054
		1172	1389	1376	1352
		1196	1394	1401	1394
		1281	1577	1580	1576
		1391	1730	1660	1667
		2809	3250	3248	3204
**HCP**	$1^1A''$	567	509	755	603
		951	975	926	1027
**HCN**	$1^1A''$	941	975	759	1053
		1496	1559	1579	1738
**C_3H_4O**	$1^1A''$	250	182	247	255
		333	341	315	313
		488	541	540	535
		582	704	547	544
		644	508	660	677
		909	1118	996	818
		1266	1611	1160	1138
		1133	1321	1406	1390
**CH_2O**	$1^1A''$	683	275	664	575
		899	1020	1077	1076
		1177	1457	1143	1089
		1321	1592	1522	1507
		2851	3228	3238	3207
		2968	3346	3375	3342
**CCl_2**	1^1B_1	303	289	273	247
		634	560	540	426
**SiF_2**	1^1B_1	252	263	247	244
		860	728	704	668
		984	828	803	771
**MEA**			**145**	**136**	**140**
**RMSE**			**187**	**176**	**172**

Table S10: Vibrational frequencies obtained using the 6-311G basis set before and after approximate projection.

System	State	Exp.	TDDFT	Δ-B3LYP	AP-Δ-B3LYP
**BH**	$1^1\Pi$	2251	2259	2462	2353
**BF**	$1^1\Pi$	1265	1061	1157	1141
**SiO**	$1^1\Pi$	853	769	754	709
**CO**	$1^1\Pi$	1518	1385	1531	1430
**N_2**	$^1\Pi$_g	1694	1622	1680	1659
**CuH**	$2^1\Sigma^+$	1698	1138	1291	1790
**Li_2**	$1^1\Sigma_u^+$	255	256	200	256
**Mg_2**	$1^1\Sigma_u^+$	191	116	188	158
**CH_2S**	1^1A_2	799	811	742	724
		820	845	823	830
		1316	1393	1368	1370
		3034	3140	3121	3111
		3081	3275	3260	3250
**C_2H_2**	1^1A_u	1048	1108	1114	1111
		1385	1398	1385	1385
**$C_2H_2O_2$**	1^1A_u	233	260	255	252
		379	386	392	379
		509	533	531	531
		720	823	816	810
		735	828	818	820
		952	1028	1032	1020
		1172	1259	1230	1172
		1196	1302	1287	1282
		1281	1412	1305	1301
		1391	1481	1464	1474
		2809	3032	3060	3035
**HCP**	$1^1A''$	567	714	705	741
		951	866	949	859
**HCN**	$1^1A''$	941	1000	1004	1009
		1496	1436	1417	1431
**C_3H_4O**	$1^1A''$	250	254	241	258
		333	304	298	298
		488	518	507	509
		582	534	532	520
		644	738	663	701
		909	964	969	970
		1266	1114	1090	1080
		1133	1369	1298	1297
**CH_2O**	$1^1A''$	683	428	564	575
		899	930	924	1076
		1177	1253	1142	1089
		1321	1369	1361	1507
		2851	3050	3020	3207
		2968	3174	3140	3342
**CCl_2**	1^1B_1	303	241	262	256
		634	458	540	498
**SiF_2**	1^1B_1	252	192	218	213
		860	542	645	614
		984	554	755	709
**MEA**			**111**	**85**	**98**
**RMSE**			**155**	**117**	**130**

Table S11: Vibrational frequencies obtained using the 6-311++G(d,p) basis set before and after approximate projection.

System	State	Exp.	CIS	Δ-HF	HF-AP-PIMOM
**BH**	$1^1\Pi$	2251	2532	2511	2332
**BF**	$1^1\Pi$	1265	1316	1311	1307
**SiO**	$1^1\Pi$	853	905	807	883
**CO**	$1^1\Pi$	1518	1636	1706	1428
**N_2**	$1^1\Pi g$	1694	1919	1894	1844
**CuH**	$2^1\Sigma_u^+ +$	1698	1810	1718	1588
**Li_2**	$1^1\Sigma_u^+ +$	255	272	245	337
**Mg_2**	$1^1\Sigma_u^+$	191	157	197	120
**CH_2S**	1^1A_2	799	862	827	816
		820	1068	894	895
		1316	1491	1491	1485
		3034	3262	3256	3239
		3081	3367	3382	3366
**$C_2H_2O_2$**	1^1A_u	233	254	248	233
		379	439	431	424
		509	547	523	525
		720	834	823	823
		735	851	840	871
		952	1015	976	969
		1172	1301	1286	1279
		1196	1326	1309	1286
		1281	1703	1680	1673
		1391	1813	1790	1805
		2809	3172	3172	3169
**HCP**	$1^1A''$	567	593	625	894
		951	1045	1004	1190
**HCN**	$1^1A''$	941	976	917	1034
		1496	1633	1555	1786
**C_3H_4O**	$1^1A''$	250	164	251	265
		333	332	313	312
		488	519	535	531
		582	676	543	543
		644	427	641	665
		909	1089	984	988
		1266	1535	1170	1153
		1133	1260	1402	1391
**CH_2O**	$1^1A''$	683	437	796	733
		899	948	1089	1109
		1177	1386	1235	1208
		1321	1632	1520	1513
		2851	3132	3211	3195
		2968	3219	3329	3310
**CCl_2**	$^1B_1$	303	312	313	316
		634	678	671	636
**SiF_2**	1^1B_1	252	290	284	279
		860	831	836	816
		984	947	951	946
**MEA**			**140**	**116**	**126**
**RMSE**			**182**	**173**	**175**

Table S12: Vibrational frequencies obtained using the 6-311++G(d,p) basis set before and after approximate projection.

System	State	Exp.	TDDFT	Δ-B3LYP	AP-Δ-B3LYP
**BH**	$1^1\Pi$	2251	2363	2510	2421
**BF**	$1^1\Pi$	1265	1224	1262	1256
**SiO**	$1^1\Pi$	853	884	881	809
**CO**	$1^1\Pi$	1518	1539	1693	1596
**N_2**	$1^1\Pi g$	1694	1737	1791	1765
**CuH**	$2^1\Sigma^+$	1698	1650	1637	1623
**Li_2**	$1^1\Sigma_u^+$	255	261	208	267
**Mg_2**	$1^1\Sigma_u^+$	191	156	191	162
**CH_2S**	1^1A_2	799	801	782	795
		820	896	836	822
		1316	1372	1351	1355
		3034	3127	3112	3101
		3081	3240	3228	3217
**C_2H_2**	1^1A_u	1048	1092	1103	1100
		1385	1433	1420	1419
**$C_2H_2O_2$**	1^1A_u	233	251	243	241
		379	386	400	392
		509	519	516	517
		720	779	758	762
		735	780	772	767
		952	971	974	965
		1172	1197	1224	1211
		1196	1239	1242	1238
		1281	1528	1426	1404
		1391	1572	1556	1564
		2809	2966	3003	2979
**HCP**	$1^1A''$	567	694	716	712
		951	957	947	947
**HCN**	$1^1A''$	941	983	985	991
		1496	1531	1509	1528
**C_3H_4O**	$1^1A''$	250	261	240	258
		333	295	292	292
		488	504	498	501
		582	508	514	502
		644	709	625	679
		909	934	941	950
		1266	1094	1087	1080
		1133	1376	1313	1307
**CH_2O**	$1^1A''$	683	575	698	634
		899	891	894	914
		1177	1300	1247	1215
		1321	1358	1301	1314
		2851	2987	2954	2973
		2968	3085	3048	3070
**CCl_2**	1^1B_1	303	192	300	301
		634	590	638	620
**SiF_2**	1^1B_1	252	233	242	240
		860	672	748	723
		984	768	861	835
**MEA**			**77**	**66**	**63**
**RMSE**			**105**	**101**	**92**

Table S13: Vibrational frequencies obtained using the aug-cc-PVDZ basis set before and after approximate projection.

System	State	Exp.	CIS	Δ-HF	HF-AP-PIMOM
**BH**	$1^1\Pi$	2251	2538	2506	2319
**BF**	$1^1\Pi$	1265	1272	1265	1265
**SiO**	$1^1\Pi$	853	859	773	767
**CO**	$1^1\Pi$	1518	1615	1645	1277
**N_2**	$1^1\Pi g$	1694	1914	1843	1842
**CuH**	$2^1\Sigma_u^+ +$	1698	1814	1722	1581
**Li_2**	$1^1\Sigma_u^+ +$	255	272	245	336
**Mg_2**	$1^1\Sigma_u^+$	191	158	199	125
**CH_2S**	1^1A_2	799	849	826	817
		820	1068	880	889
		1316	1475	1474	1473
		3034	3273	3264	3241
		3081	3384	3394	3372
**$C_2H_2O_2$**	1^1A_u	233	255	247	234
		379	436	428	422
		509	542	519	523
		720	831	826	821
		735	850	838	872
		952	1025	990	989
		1172	1284	1273	1268
		1196	1309	1291	1268
		1281	1700	1664	1655
		1391	1800	1772	1783
		2809	3180	3179	3118
**HCP**	$1^1A''$	567	593	583	759
		951	1045	950	1082
**HCN**	$1^1A''$	941	965	905	1008
		1496	1625	1536	1768
**C_3H_4O**	$1^1A''$	250	160	250	264
		333	331	312	312
		488	516	531	529
		582	671	541	541
		644	413	627	651
		909	1076	978	982
		1266	1527	1165	1148
		1133	1245	1391	1380
**CH_2O**	$1^1A''$	683	449	798	729
		899	934	1075	1096
		1177	1368	1226	1198
		1321	1627	1496	1490
		2851	3143	3218	3201
		2968	3238	3343	3322
**CCl_2**	${}^1B_1$	303	309	307	309
		634	683	669	635
**SiF_2**	1^1B_1	252	280	274	266
		860	819	823	808
		984	932	934	935
**MEA**			**136**	**109**	**119**
**RMSE**			**183**	**168**	**166**

Table S14: Vibrational frequencies obtained using the aug-cc-PVDZ basis set before and after approximate projection.

System	State	Exp.	TDDFT	Δ-B3LYP	AP-Δ-B3LYP
**BH**	$1^1\Pi$	2251	1754	2492	2397
**BF**	$1^1\Pi$	1265	1190	1231	1224
**SiO**	$1^1\Pi$	853	884	881	766
**CO**	$1^1\Pi$	1518	1522	1673	1574
**N_2**	$1^1\Pi g$	1694	1741	1793	1767
**CuH**	$2^1\Sigma^+$	1698	1647	1650	1780
**Li_2**	$1^1\Sigma_u^+$	255	261	261	268
**Mg_2**	$1^1\Sigma_u^+$	191	80	191	163
**CH_2S**	1^1A_2	799	789	770	784
		820	898	840	827
		1316	1356	1336	1341
		3034	3132	3117	3105
		3081	3254	3240	3228
**C_2H_2**	1^1A_u	1048	1090	1101	1099
		1385	1432	1419	1419
**$C_2H_2O_2$**	1^1A_u	233	250	242	239
		379	382	395	387
		509	515	512	513
		720	780	763	763
		735	782	773	772
		952	984	989	977
		1172	1181	1209	1198
		1196	1227	1231	1226
		1281	1521	1418	1400
		1391	1566	1550	1561
		2809	2974	3012	2979
**HCP**	$1^1A''$	567	700	711	714
		951	943	933	935
**HCN**	$1^1A''$	941	975	973	979
		1496	1524	1501	1522
**C_3H_4O**	$1^1A''$	250	262	241	258
		333	295	292	291
		488	505	496	499
		582	508	517	505
		644	721	625	680
		909	931	940	950
		1266	1088	1082	1076
		1133	1369	1313	1308
**CH_2O**	$1^1A''$	683	544	682	619
		899	874	878	898
		1177	1279	1248	1218
		1321	1355	1273	1288
		2851	2995	2955	2975
		2968	3100	3056	3078
**CCl_2**	1^1B_1	303	192	294	295
		634	590	645	627
**SiF_2**	1^1B_1	252	223	237	233
		860	662	737	712
		984	758	846	822
**MEA**			**81**	**66**	**65**
**RMSE**			**111**	**100**	**92**

Table S15: Vibrational frequencies obtained using the aug-cc-PVTZ basis set before and after approximate projection.

System	State	Exp.	CIS	Δ-HF	HF-AP-PIMOM
**BH**	$1^1\Pi$	2251	2545	2522	2430
**BF**	$1^1\Pi$	1265	1366	1363	1358
**SiO**	$1^1\Pi$	853	937	823	818
**CO**	$1^1\Pi$	1518	1632	1676	1323
**N_2**	$1^1\Pi g$	1694	1899	1873	1817
**CuH**	$2^1\Sigma_u^+ +$	1698	1814	1570	1586
**Li_2**	$1^1\Sigma_u^+ +$	255	273	243	334
**Mg_2**	$1^1\Sigma_u^+$	191	156	194	107
**CH_2S**	1^1A_2	799	847	830	823
		820	1070	882	889
		1316	1487	1490	1487
		3034	3262	3261	3243
		3081	3362	3383	3366
**$C_2H_2O_2$**	1^1A_u	233	257	251	237
		379	439	431	425
		509	544	520	525
		720	839	833	826
		735	859	845	876
		952	1009	972	974
		1172	1296	1281	1276
		1196	1327	1307	1285
		1281	1709	1674	1665
		1391	1805	1787	1800
		2809	3162	3162	3116
**HCP**	$1^1A''$	567	509	593	791
		951	975	985	1080
**HCN**	$1^1A''$	941	976	861	1033
		1496	1633	1671	1765
**C_3H_4O**	$1^1A''$	250	166	253	268
		333	333	314	314
		488	519	535	532
		582	679	546	546
		644	437	654	676
		909	1089	989	994
		1266	1638	1169	1152
		1133	1257	1405	1395
**CH_2O**	$1^1A''$	683	437	796	733
		899	948	1089	1109
		1177	1386	1235	1208
		1321	1632	1520	1513
		2851	3132	3211	3195
		2968	3132	3329	3310
**CCl_2**	$^1B_1$	303	311	311	316
		634	676	666	635
**SiF_2**	1^1B_1	252	464	291	282
		860	831	867	850
		984	899	986	987
**MEA**			**144**	**119**	**124**
**RMSE**			**182**	**177**	**169**

Table S16: Vibrational frequencies obtained using the aug-cc-PVTZ basis set before and after approximate projection.

System	State	Exp.	TDDFT	Δ-B3LYP	AP-Δ-B3LYP
**BH**	$1^1\Pi$	2251	2395	2520	2348
**BF**	$1^1\Pi$	1265	1274	1301	1295
**SiO**	$1^1\Pi$	853	896	896	817
**CO**	$1^1\Pi$	1518	1547	1687	1582
**N_2**	$1^1\Pi g$	1694	1730	1873	1751
**CuH**	$2^1\Sigma^+$	1698	1664	1656	1793
**Li_2**	$1^1\Sigma_u^+$	255	260	209	267
**Mg_2**	$1^1\Sigma_u^+$	191	157	191	161
**CH_2S**	1^1A_2	799	787	770	784
		820	902	845	830
		1316	1368	1349	1353
		3034	3130	3118	3109
		3081	3237	3227	3219
**C_2H_2**	1^1A_u	1048	1093	1102	1099
		1385	1436	1424	1424
**$C_2H_2O_2$**	1^1A_u	233	250	244	243
		379	382	401	393
		509	515	516	518
		720	780	769	765
		735	782	776	776
		952	984	977	969
		1172	1181	1227	1215
		1196	1227	1243	1239
		1281	1521	1422	1401
		1391	1566	1551	1560
		2809	2974	3003	2975
**HCP**	$1^1A''$	567	691	705	700
		951	958	949	950
**HCN**	$1^1A''$	941	990	988	991
		1496	1528	1507	1525
**C_3H_4O**	$1^1A''$	250	264	242	259
		333	296	293	293
		488	509	500	503
		582	510	519	507
		644	721	635	686
		909	939	951	958
		1266	1092	1088	1081
		1133	1380	1315	1310
**CH_2O**	$1^1A''$	683	567	689	635
		899	885	891	912
		1177	1300	1251	1217
		1321	1358	1300	1313
		2851	2991	2960	2979
		2968	3085	3050	3072
**CCl_2**	1^1B_1	303	270	299	300
		634	628	639	623
**SiF_2**	1^1B_1	252	243	253	249
		860	710	772	750
		984	823	886	866
**MEA**			**73**	**68**	**62**
**RMSE**			**102**	**103**	**85**

2 Geometries

The XYZ coordinates can be found on figshare.com under the folder PIMOM_Vibrational_XYZ.zip: `https://doi.org/10.6084/m9.figshare.21507105.v1`.
